# Hypermethylation of CDKN2A CpG island drives resistance to PRC2 inhibitors in SWI/SNF loss-of-function tumors

**DOI:** 10.1038/s41419-024-07109-3

**Published:** 2024-11-05

**Authors:** Xinghao Wang, Yajun Wang, Min Xie, Shichao Ma, Yilin Zhang, Lele Wang, Yangfeng Ge, Guobin Li, Mengxi Zhao, Sheng Chen, Chenxi Yan, Hailong Zhang, Wei Sun

**Affiliations:** 1 Department of Thoracic Surgery, Tongji Hospital, Huazhong University of Science and Technology, Wuhan, China; 2 Blueray Biopharma Inc., Shanghai, China

**Keywords:** Cancer therapeutic resistance, Predictive markers

## Abstract

Polycomb repressive complex 2 (PRC2) catalyzes the writing of the tri-methylated histone H3 at Lys27 (H3K27me3) epigenetic marker and suppresses the expression of genes, including tumor suppressors. The function of the complex can be partially antagonized by the SWI/SNF chromatin-remodeling complex. Previous studies have suggested that PRC2 is important for the proliferation of tumors with SWI/SNF loss-of-function mutations. In the present study, we have developed an EED-directed allosteric inhibitor of PRC2 termed BR0063, which exhibits anti-proliferative properties in a subset of solid tumor cell lines harboring mutations of the SWI/SNF subunits, SMARCA4 or ARID1A. Tumor cells sensitive to BR0063 exhibited several distinct phenotypes, including cell senescence, which was mediated by the up-regulation of *CDKN2A*/p16. Further experiments revealed that the expression of p16 was suppressed in the BR0063-resistant cells via DNA hypermethylation in the CpG island (CGI) promoter region, rather than via PRC2 occupancy. The expression of *TET1*, which is required for DNA demethylation, was found to be inversely correlated with p16 CGI methylation, and this may serve as a biomarker for the prediction of resistance to PRC2 inhibitors in SWI/SNF LOF tumors.

## Introduction

Polycomb repressive complex 2 (PRC2) is a large multimeric enzymatic complex that functions in chromatin regulation, catalyzing the mono-, di- and tri-methylation of lysine 27 on histone H3 (H3K27), which acts as a marker for the repression of genes, including those encoding key developmental regulators [1]. PRC2 complex comprises three core subunits, EZH2, EED, and SUZ12, which form a 1:1:1 complex. EZH2 is the catalytic subunit of the complex, which contains the SET domain required for the methyltransferase activity of PRC2 [2]. EED not only serves as a scaffold protein that bridges EZH2 and SUZ12, but it also functions as a histone reader that recognizes the H3K27me3 marker. Such reader function is important for both the allosteric activation of PRC2 [3] and the expansion of this suppressive marker along the chromatin template [4]. Although PRC2 function is essential for normal developmental processes, its overactivation has been shown to contribute to the development of multiple tumors. EZH2 overexpression was found to be associated with heightened aggressiveness of cancer and the advanced disease state of solid tumors in a wide range of different cancer types, including prostate, breast, bladder, and endometrial cancers [5–7]. Moreover, the gain-of-function mutations of EZH2 were identified in a subset of patients with diffuse large B-cell lymphoma and follicular lymphoma [8, 9]. PRC2 inhibitors, such as the EZH2 inhibitor tazemetostat (EPZ-6438), have been clinically approved for the treatment of such malignancies [10].

The SWI/SNF chromatin-remodeling complex utilizes ATP hydrolysis to alter the chromatin state, thereby creating a more open and accessible chromatin structure for transcriptional activation. The SWI/SNF complex is generally considered to be a tumor suppressor, and its loss-of-function (LOF) mutations have been found in ~20% of all tumors, which are associated with tumor progression [11]. A previous study [12] demonstrated that the PRC2 and SWI/SNF complexes functionally antagonize each other during the processes of gene expression regulation, development, and cancer. Tumors with LOF mutations of the AT-interacting domain-rich protein 1 A (ARID1A) or SWI/SNF-related, matrix-associated, actin-dependent regulator of chromatin (SMARC), subfamily a, member 4 (SMARCA4), the two most frequent mutated SWI/SNF subunits in solid tumors, may benefit from PRC2 inhibition [13–15]. However, at present, the approval of PRC2 inhibitors in treating SWI/SNF LOF tumors in the clinic has been limited to rare epithelioid sarcoma with the SMARCB1 LOF mutation [16, 17]. Progress in the further application of PRC2 inhibitors in solid tumors has also been hampered by low response rates and a lack of precise biomarkers for the prediction of drug sensitivity [18].

To further study the application of PRC2 inhibitors in solid tumors containing LOF mutations in ARID1A or SMARCA4, endometrium, lung, ovary and gastrointestinal tumor cell lines bearing LOF mutations in either of the subunits were selected in the present study to investigate the effects of BR0063, our in-house-developed PRC2 allosteric inhibitor, on the inhibition of PRC2 activity, as well as on cell proliferation in response to the treatment. BR0063 was found to efficiently block the interaction of the EED subunit with H3K27me3, which led to the marked inhibition of cellular PRC2 activity. However, the data obtained in the present study, in agreement with data from the public database DepMap, indicated that only a subset of the SWI/SNF LOF tumor cells were sensitive to PRC2/EED perturbation. To investigate the underlying mechanism of resistance to PRC2 inhibition, genes that were found to be differentially expressed upon treatment with BR0063 in sensitive and resistant cells were profiled by RNA sequencing (RNA-Seq), and the associated pathways were analyzed. The specific up-regulation of cyclin-dependent kinase inhibitor 2 A (*CDKN2A*)/p16INK4a (denoted as ‘p16’ for short), and the down-regulation of E2F genes, were identified in sensitive cells, whereas the expression of p16 was suppressed in the resistant cells via CGI promoter hypermethylation. Interestingly, the expression of the DNA demethylase ten-eleven translocation methylcytosine dioxygenase 1 (*TET1*) appeared to correlate with the CGI methylation status of p16, and consequently, this may be used as a biomarker for the prediction of an improved response of SWI/SNF LOF tumors to PRC2/EED inhibition.

## Materials and methods

### AlphaScreen assay

BR0063 was serial-diluted (threefold) in the binding buffer [25 mM HEPES (pH 8.0), 50 mM NaCl, 0.5% BSA (cat. no. # B2064; Sigma-Aldrich, St. Louis, MO, USA) and 0.02% Tween-20] and added into each well of the ProxiPlate (PerkinElmer, Inc., Waltham, MA, USA) with brief centrifuging. His-EED protein solution (final concentration, 15 nM, in-house produced) and biotin-H3K27me3 peptide solution (final concentration, 18.75 nM, synthesized by GenScript, Piscataway, NJ, USA) were also prepared in the same buffer and added sequentially into the plate in order. The plate was then incubated for 30 min at room temperature (RT) while the AlphaScreen beads mixture (from the AlphaScreen Histidine Detection Kit; cat. no. #6760619 C; PerkinElmer, Inc.) was prepared, following the manufacturer’s protocol. The AlphaScreen beads mixture was subsequently added into each well of the ProxiPlate, and the plate was then sealed with aluminum foil. The plate was then centrifuged at 600 rev./min for 10 sec, after which the plate was incubated at RT for 1 h, and the AlphaScreen signals were then detected by the EnVision microplate reader (PerkinElmer, Inc.). All experiments were performed in duplicate to confirm reproducibility, and the data were fitted using Prism (Dotmatics, Boston, MA, USA) according to the formula: [Inhibitor] vs. response—Variable slope (four parameters) model and the EC_50_ values were then calculated.

### Cell culture

DMS 114, NCI-H23, NCI-H1703, HEC-1-A, EFE-184 and NUGC-3 cells were cultured in Gibco^®^ RPMI-1640 medium (ATCC modified; cat. no. #A1049101; Thermo Fisher Scientific, Inc., Waltham, MA, USA) with 10% Gibco^®^ FBS (cat. no. #10091148; Thermo Fisher Scientific, Inc.). The MFE-296, HuTu-80, TOV-112D, and HEK293T cells were cultured in Gibco^®^ DMEM medium (cat. no. #11995073; Thermo Fisher Scientific, Inc.) containing 10% FBS. All cell lines were purchased from Cobier Bioscience (Nanjing, China) with STR validation. All cells were routinely checked for mycoplasma contamination using MycoAlert^®^ Mycoplasma Detection Kit (cat. no. #LT07-318; Lonza Bioscience, Basel, Switzerland) to ensure that the cells were mycoplasma-free.

### ELISA

Cells were diluted to the appropriate concentration and subsequently were passaged into a 96-well flat-bottom plate (cat. no. #3599; Corning, Inc., Corning, NY, USA) in a cell-culture medium containing 5% FBS to allow cells to reach a state of near-confluence at day 3. BR0063 (diluted in DMSO; Hybri-Max™; cat. no. #D2650; Sigma-Aldrich, final concentration of 1 μM with 3× serial dilution) or DMSO control was added to cultured cells, with subsequent incubation in cell incubator for 3 days. Cells were then washed with PBS (Gibco^®^; cat. no. #10010049; Thermo Fisher Scientific, Inc.), and 100 μl of lysis buffer (containing 0.4 M HCl) was added into each well, with a further incubation for at least 30 min at RT. Subsequently, 80 μl of neutralizing buffer (0.5 M Na_2_HPO_4_ with protease inhibitor cocktail; cat. no. #P8340; Sigma-Aldrich) was added and mixed well with the cell lysate. The ELISA plate (cat. no. #40303; BEAVER, Suzhou, China) was then coated with 30 μl (for H3K27me3 detection) or 3 µl (for histone H3 detection) of cell lysate diluted with PBS to a final volume of 100 μl in each well at 4 °C overnight. Subsequently, 5% BSA solution with TBST (150 μl) was added to each well as a blocking agent and incubated at RT for 1.5 h, after which the wells were washed with 300 μl of TBST 5 times. Aliquots of primary antibody [100 μl; histone H3 rabbit polyclonal antibody: cat. no. CST#9715, dilution, 1:4000; or tri-methyl-histone H3 (Lys27) rabbit monoclonal antibody: cat. no. CST#9733, dilution, 1:1000; CST Biological Reagents Co., Ltd., Danvers, MA, USA] were added, and the mixture was incubated at RT for 1 h. The primary antibody was then removed, and the wells were washed 5 times with 300 μl of TBST. HRP-conjugated secondary antibody [100 μl; goat anti-rabbit IgG, H&L (HRP): cat. no. #ab205718, dilution, 1:4000; Abcam, Cambridge, MA, USA] was then added, and the mixture was incubated at RT for 1 h. After washing the wells with 300 μl of TBST 3 times, 100 μl of TMB substrate A + B (Sangon Biotech, Shanghai, China) was added to the well, and incubated for 2-10 min at RT. Subsequently, 50 μl of stop solution (2 M H_2_SO_4_) was added, and the absorption signal was read at 450 nm. All experiments were performed in duplicate to confirm reproducibility. The relative H3K27me3 level was calculated according to the following formula: [Abs(H3K27me3)_compound_/Abs(total H3)_compound_)/(Abs(H3K27me3)_DMSO_/Abs(total H3)_DMSO_]. The data were processed using Prism (Dotmatics) according to the formula: [Inhibitor] vs. response—Variable slope (four parameters) model, and finally, the EC_50_ values were calculated.

For ELISA detection in tumor tissue samples, ~30 mg of snap-freezing tissue in 400 μl of lysis buffer was used, with homogenization in a tissue homogenizer. The samples were centrifuged at 16,000 × *g* for 10 min while the temperature were kept at 4 °C, the supernatant was collected and neutralized with 320 μl of neutralizing buffer. The remainder of the procedures are the same as those described above for cell ELISA. All of samples in the vehicle group, and the first 7 out of 8 samples in the BR0063-treatment groups were included in the experiment due to limited well number in one 96-well ELISA plate. Each sample were duplicated.

### Cell viability assay

Cells were diluted in cell culture medium containing 5% FBS to the appropriate concentration, and were then passaged into a 96-well plate (cat. no. #3904; Corning, Inc.) to allow the cells to reach near-confluence at day 7. BR0063 at a final concentration of 1 μM with 3× serial dilution, or DMSO as a control, was added to the wells with further incubation for 7 days. The culture medium was then removed, and the cells were digested using Gibco^®^ Trypsin-EDTA (cat. no. #25200056; Thermo Fisher Scientific, Inc.) and then resuspended in the well. Corresponding percentages of cells were then passaged into a new 96-well cell culture plate with fresh culture medium and series of dilutions of BR0063, and the cells were further incubated for 7 days (total 14 days of compound incubation). CellTiter-Glo^®^ reagent (1:1; cat. no. #G7573; Promega Corporation, Madison, WI, USA) was added into each well, mixed well, and incubated for 10 min at RT to permit the luminescent signal to be read. All experiments were performed in triplicate to confirm reproducibility. The data were normalized against that of the DMSO control, and fitted with the formula: [Inhibitor] vs. response—Variable slope (four parameters) using Prism software (Dotmatics, USA) for calculation of the EC_50_ values.

### DepMap data analysis

All data were downloaded from DepMap website (https://depmap.org/). The EED dependency was based on the published RNAi data [19, 20] normalized by DEMETER2 [21]. SWI/SNF LOF was defined by the LOF mutations in either ARID1A or SMARCA4. For cells harboring SMARCA4 LOF mutations, only those with low expression of SMARCA2 were counted as SWI/SNF LOF, according to previous reports [14, 15].

### RNA-Seq sample preparation and data analysis

Selected SWI/SNF LOF tumor cells were cultured in the cell-culture medium with or without 1 μM BR0063 for 6 days in 25 cm^2^ cell-culture flasks (cat. no. #430639; Corning, Inc.). Cells were collected, washed once with PBS, and stored on dry ice prior to RNA extraction. Quality control of the extracted RNA was performed prior to sequencing to ensure the quality of RNA. All experiments were performed in duplicate to minimize the possible deviations. Sequencing was performed by Genewiz, Inc. (Suzhou, China) using an Illumina^®^ Hiseq/Novaseq platform (Illumina, Inc.) with a sequencing depth of 6 G per sample. Total pair-end sequences obtained (2 × 150 bp) were aligned with the human genome (hg19). The mRNA levels of genes were calculated as Fragments Per Kilobase Million (FPKM). Differential gene expression was determined using R package DESeq2 [22], based on read counts. Differently expressed genes were selected with a false discovery rate (FDR) threshold of 0.05, and a Log2 fold change (log_2_FC) threshold of ±0.585 (1.5-fold). The overlapping genelist was analyzed using a Venn diagram by jvenn [23] (http://jvenn.toulouse.inra.fr/app/example.html), and KEGG pathway analysis was performed using KOBAS [24] [web application (http://kobas.cbi.pku.edu.cn/genelist/)]. Gene set enrichment analysis (GSEA) [25] was performed using a GSEA desktop application (downloaded from http://www.gsea-msigdb.org/gsea/downloads.jsp). Data visualization was performed using the R packages, pheatmap and ggplot. The RNA-Seq raw data have been deposited into the CNGB Sequence Archive (CNSA) [26] of the China National GeneBank DataBase (CNGBdb) [27] with accession number CNP0005260.

*Cell cycle analysis by PI staining*. Cells were treated with or without 1 μM BR0063 for 7 days, digested using Gibco^®^ Trypsin-EDTA (cat. no. #25200056; Thermo Fisher Scientific, Inc.) and centrifuged at 250×g to collect the cell pellet. Subsequently, the cells were resuspended in pre-chilled 70% ethanol and stored at −20 °C overnight for fixation. The fixed cells were then washed with cold PBS twice, collected by spinning at 500 g, resuspended with 500 μL of Invitrogen^®^ FxCycle™ PI/RNase Staining Solution (cat. no. #F10797; Thermo Fisher Scientific, Inc.) containing RNase A, and incubated for 30 min at RT. The stained cell samples were then filtered through a 35-μm cell strainer (Falcon®; cat. no. #352235; Corning, Inc.), and the PI-stained signals were detected using a CytoFLEX™ flow cytometer (Beckman Coulter, Inc., Brea, CA, USA) using the PE channel. Finally, cell cycle data analysis was performed using Watson Pragmatic algorithm [28] in FlowJo 10.10 univariate cell cycle platforms (BD Biosciences, San Jose, CA, USA). The significance of differences of the cell cycle data were determined using two-way ANOVA by Prism software (Dotmatics, USA).

### Senescence β-galactosidase staining

Cell senescence status was detected using a Senescence β-galactosidase Staining Kit (cat. no. CST#9860; CST Biological Reagents Co., Ltd.). Briefly, cells were cultured in a 24-well plate (cat. no. #3524; Corning, Inc.) with medium with or without 1 μM BR0063 for 10 days (with the exception of 6 days’ culture for HuTu-80 cells); subsequently, the medium was removed and the plate was rinsed one time with 1×PBS. 1X Fixative Solution (from the kit) was then added to each well. The cells were then incubated for 10-15 min at RT to allow fixation. Subsequently, the plate was rinsed twice with 1×PBS, and 250 μl of the β-galactosidase Staining Solution (from the kit) was added to each well. The plate was then sealed with parafilm to prevent evaporation, and the plate was incubated at 37 °C for 2 days in a CO_2_-free incubator, and the cell senescence were indicated by the development of blue color under the microscope.

### Caspase-3/-7 assay

DMS 114 and MFE-296 cells were passaged into a 6-well plate (cat. no. #3516; Corning, Inc.) and incubated with or without 1 μM BR0063 for 7 days. Cells were also prepared for incubation with 10 μM oxaliplatin (cat. no. #HY-17371; MCE, Monmouth Junction, NJ, USA) for 2 days. The incubation time for BR0063 in TOV-112D and HuTu-80 cells was adjusted to 10 and 6 days respectively, whereas the incubation time for cells in the presence of oxaliplatin was adjusted to 1 day. All tested groups have the final DMSO concentration of 0.1% in the cell culture medium. The cellular caspase activity was measured using a Caspase-Glo^®^ 3/7 Assay (cat. no. G8981; Promega Corporation) at the end of incubation. Caspase-Glo^®^ 3/7 reagent was freshly prepared by dissolving lyophilized Caspase-Glo^®^ 3/7 Substrate into Caspase-Glo^®^ 3/7 Buffer. The reagent was added into each well in a 1:1 ratio, followed by incubation at RT in dark for 30 min-1 h; The cell viability for in parallel incubated cell samples were also determined using CellTiter-Glo^®^ reagent (1:1). The relative Caspase-3/7 activity was determined by the intensity of luminescent signal normalized to control samples as well as cell viability. All experiments were performed at least in triplicate to confirm reproducibility.

### Generation of stable cell lines with doxycycline-inducible p16 knockdown

HEK293T cells were co-transfected with plasmids of a lentiviral vector with Tet-inducible shRNA expression (modified based on Addgene, cat. no. #21915; Addgene, Inc., Cambridge MA, USA), packaging plasmid (psPAX2, same with Addgene cat. no. #12260) and envelope plasmid (pMD2.G, same with Addgene cat. no. #12259) using Invitrogen^®^ Lipofectamine™ 3000 Transfection Reagent (cat. no. #L3000008; Thermo Fisher Scientific, Inc.). The supernatant containing the lentivirus was collected 48 and 72 h after transfection, followed by filtering through a 0.45 μM filter. The freshly collected lentivirus was used to infect the target DMS 114 and MFE-296 cells in the presence of 8 μg/ml polybrene (cat. no. #TR-1003; Sigma-Aldrich). At 72 h post-transduction, 1 µg/ml puromycin (Gibco^®^ cat. no. #A1113803; Thermo Fisher Scientific, Inc.) was added into cell-culture medium of both transduced cells and parental cells serving as a control. The cells were then incubated for a further 7 days, until all control cells had died. The remaining transduced cells were then passaged into the fresh cell-culture medium w/o puromycin for further experiments. The sequence of the shRNA targeting CDKN2A/p16 was 5’-CACTACCGTAAATGTCCATTT-3’.

### Reverse-transcription (RT)-qPCR analysis

Total RNA samples were extracted using E.Z.N.A.^®^ HP Total RNA Kit (cat. no. #R6812-02; Omega Bio-Tek, Inc., Norcross, GA, USA) following the manufacturer’s protocol. cDNA was prepared by reverse transcription using 0.5 µg of total RNA and iScript Reverse™ Transcription Supermix (cat.no. #1708841; BioRad Laboratories, Inc., Hercules, CA, USA). qPCR analysis was performed using iTaq™ Universal SYBR Green Supermix (cat. no. #1725121; BioRad Laboratories, Inc.) on a CFX Real-Time System (BioRad Laboratories, Inc.) following product protocol. β-actin was used as the normalization control, and the relative gene expression levels were calculated using the 2-^ΔΔCt^ method [29]. All reactions were performed in triplicate. The sequence information for the primers is provided in the Supplementary Table S2.

### Western blotting (WB)

Cells were washed once with cold PBS, and then covered by WB Lysis Buffer (10 mM Tris/HCl, pH 8.0/1% SDS), and the cells resuspended in WB Lysis Buffer were collected into a 1.5 mL centrifuge tube and heated at 95 °C for 10 min. Whole cell protein was obtained by centrifuging the solution at 16,000 × *g* for 10 min to collect the supernatant. The protein concentration was then determined using Pierce™ BCA Protein Assay Kits (cat. no. #23225, Thermo Scientific, Inc.), and ~20-40 µg of whole cell protein was applied for SDS-PAGE electrophoresis using SurePAGE™ 4-12% Bis-Tris gel (cat. no. #M00653, GenScript, Piscataway, NJ, USA). Subsequently, the proteins on gel were transferred to a PVDF membrane (0.22 μm, cat. no. #L00735C, GenScript) via the wet transfer method using an eBlot™ L1 Fast Wet Transfer System (GenScript), following the manufacturer’s protocol. The membrane was blocked with TBST with 5% BSA, and subsequently incubated with primary antibodies [SMARCA4 rabbit mAb: cat. no. CST#49360, dilution, 1:500; ARID1A rabbit mAb: cat. no. CST#12354, dilution, 1:500; histone H3 rabbit polyclonal antibody: cat. no. CST#9715, dilution, 1:1000; tri-methyl-histone H3 (Lys27) rabbit monoclonal antibody: cat. no. CST#9733, dilution, 1:1000; β-actin rabbit mAb: cat. no. CST#8457, dilution, 1:1000; Rb rabbit mAb: cat. no. CST#9313, dilution 1:500; phospho-Rb (Ser780) rabbit mAb: cat. no. CST#8180, dilution 1:1000; all antibodies provided by CST Biological Reagents Co., Ltd.] diluted in SuperBlock™ Blocking Buffer (cat. no. #37535; Thermo Scientific, Inc.) overnight at 4 °C, and secondary antibody [goat anti-rabbit IgG H&L (HRP): cat. no. #ab205718, dilution 1:4000; Abcam) for 1 h at RT. ECL Western Blotting Substrate (cat. no. #34577; SuperSignal™ West Pico; Thermo Scientific, Inc.) was applied to the membrane for signal development, and images were acquired by ChemDoc^TM^ Imaging System (BioRad Laboratories, Inc.) using the chemiluminescence mode.

### Chromatin immunoprecipitation (ChIP)-qPCR assay

Cells were incubated with or without 1 μM BR0063 for 3 days. For ChIP sample preparation, cells were cross-linked with 1% formaldehyde (cat. no. #F8775; Sigma-Aldrich) at RT for 10 min. The formaldehyde was then quenched by adding Glycine stock solution from SimpleChIP^®^ Enzymatic Chromatin IP Kit (cat. no. #9003; Magnetic Beads; CST Biological Reagents Co., Ltd.) and incubated for 5 minutes at RT. Cross-linked cell was collected using cold plastic cell scraper, centrifuged at 2000×g and washed with PBS to remove the medium. Chromatin extraction was performed using the same kit following the manufacturer’s instructions. Specifically, 0.45 μl (for the DMS 114 and MFE-206 cells), 0.6 μl (for the Hutu-80 and TOV-112D cells) and 0.75 μl (for the NCI-H23 cells) of micrococcal nuclease (MNase, provided in the Chromatin IP kit) was added to the nuclei of each immunoprecipitate (4×10^6^ cells) to obtain optimal chromatin size. For chromatin immunoprecipitation, antibodies against H3K27me3 (cat. no. CST#9733; dilution, 1:50), EZH2 (cat. no. CST#5246; dilution, 1:100) and normal rabbit IgG (supplied with the kit as negative control) were incubated with the chromatin extract overnight, and the bound protein, as well as cross-linked DNA were immunoprecipitated using ChIP-Grade Protein G Magnetic Beads. Proteins were removed by protease A digestion, and DNA samples were purified using DNA spin column provided in the kit. The purified DNA was then subjected to qPCR analysis. All reactions were performed in triplicate. The sequence information for the primers is shown in the Supplementary Table S2.

### Methylation-specific PCR analysis

Genomic DNA was purified using an Invitrogen^®^ PureLink™ Genomic DNA Mini Kit (cat. no. #K182002; Thermo Scientific, Inc.), and processed by EpiJET Bisulfite Conversion Kit (cat. no. #K1461; Thermo Scientific, Inc.) using 500 ng of genomic DNA. PCR amplification was performed using an Applied Biosystems^®^ AmpliTaq Gold™ 360 Master Mix (cat. no. #4398881; Thermo Fisher Scientific, Inc.) with methylation-specific primers designed for the p16 CGI promoter region for 40 cycles. The PCR products were analyzed using 2% agar gels with YeaRed (cat. no. #10202ES76; YEASEN, Shanghai, China) staining. The sequence information for the primers is listed in the Supplementary Table S2.

### Establishment of the cell-derived xenograft model in mice

In vivo efficacy studies of BR0063 in DMS 114 and MFE-296 xenograft models were performed by subcutaneous injection of 5 × 10^6^ cells mixed with Matrigel™ (cat. no. #354234; Corning, Inc., Corning, NY, USA) into female BALB/c nude mice (6–10 weeks old, purchased from Vital River, Beijing, China; *n* = 8 for each group). Tumor-bearing mouse were then randomized when the tumor volume reached 80-120 mm^3^, and treated with BR0063 (10, 30, or 80 mg/kg, orally, twice daily, abbreviated as “mpk, po bid”). Tumor volumes and body weights of the inoculated mice were subsequently measured every 3–4 days. The animal studies were performed by Shanghai Medsyin Biopharma Co., Ltd in the animal facility of Shanghai Institute for Biomedical and Pharmaceutical Technologies (SIBPT). The maximal tumor size/burden permitted is 3000 mm^3^ for single mice or 2000 mm^3^ for the average tumor volume in each study group. The maximal tumor size/burden permitted was not exceeded. All animal studies were carried out following the SOP of SIBPT animal facility.

### IHC staining

Pieces of the tumor samples were fixed in 10% formaldehyde overnight, and paraffin-embedded for the IHC staining of p16 and Ki67. IHC staining was performed by Hubei BIOSSCI Biotechnology Co., Ltd. (Hubei, China) with anti-CDKN2A/p16INK4a antibody (cat. no. ab108349; 1:6,400 dilution; Abcam) and anti-Ki67 antibody (cat. no. ab16667; 1:100 dilution; Abcam), and counterstained with hematoxylin. Whole IHC slides were scanned under a high-throughput digital slide scanner (NanoZoomer S360; Hamamatsu, Iwata City, Japan). For each IHC slide, four ×40 objective magnification fields (avoiding the necrotic areas) were analyzed using Image-Pro Plus software (Media Cybernetics, Rockville, MD, USA). For quantification of the Ki67 positivity rates, the ratios of DAB-positive cells to total cells were counted; for quantification of the p16 positivity rates, the ratios of the DAB-positive area to total cell area were counted. Samples with dominant necrotic areas were excluded in the quantification analysis, left 7 out of 8 samples in each treatment group available for the statistical analysis.

### Statistical analysis

The graphical data in vitro are shown as the mean ± standard deviation (SD), whereas the in vivo data are shown as the mean ± standard error of the mean (SEM). The significance of differences of the animal experiment data were determined using unpaired Student’s t tests (for comparisons of 2 groups) or one-way ANOVA (for comparisons of ≥2 groups) by Prism software (Dotmatics, USA). Statistically significant values are indicated as follows: *p < 0.05; **p < 0.01; ***p < 0.001; and ****p < 0.0001. The statistical analysis data for ELISA and the IHC data for tumor tissues are presented as scatterplots, with the bars representing the median values.

## Results

### BR0063 inhibits cellular PRC2 activity and blocks proliferation in a subset of SWI/SNF LOF tumor cell lines

**Fig. 1:**
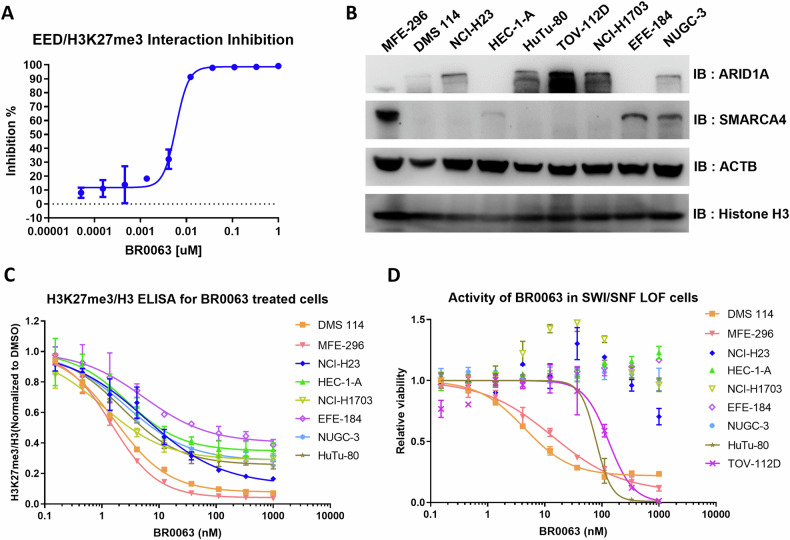
BR0063 demonstrates good in vitro activity, and efficiently inhibits PRC2 in SWI/SNF LOF mutation cell lines. **A** AlphaScreen assay detection of the inhibitory effect of BR0063 on the interaction of EED with the H3K27me3 peptide. Each experimental condition was performed in duplicate, and error bars are presented as the mean ± SD. **B** Western blotting (WB) detection of the SWI/SNF subunits ARID1A and SMARCA4 in selected SWI/SNF mutated cell lines. **C** ELISA detection of H3K27me3 and Histone H3 in cells incubated with serial dilution of BR0063 for 3 days, normalized with DMSO control. Each experiment was performed in duplicate, and error bars are shown as the mean ± SD. **D** CellTiter-Glo^®^ detection of cell viability of cells incubated with serial dilution of BR0063 for 14 days, normalized with DMSO control. The calculated EC50 values for DMS 114, MFE-296, HuTu-80, and TOV-112D cells are 4.4, 13.7, 79.3, and 132 nM, respectively. Each experiment was performed in triplicate, and the error bars represented the mean ± SD.

Initially, we developed a potent and selective PRC2 allosteric inhibitor termed BR0063, which was found to block the EED-H3K27me3 interaction in vitro with a half maximal effective concentration (EC_50_) of 5.9 nM, as determined from the AlphaScreen assay (Fig. 1A). A panel of endometrium, lung, ovary and gastrointestinal tumor cell lines with SWI/SNF LOF mutations (Supplementary Table S1) were selected, and the expression levels of the SWI/SNF subunits were analyzed by western blotting (Fig. 1B). LOF mutations of ARID1A or SMARCA4, the two most frequently mutated SWI/SNF subunits in solid tumors [30], led to the absence of protein expression in almost all tested cell lines, and this finding was consistent with that of previous reports [14, 15]. One exception is NUGC-3, although with positive expression of ARID1A, the product resulted is a non-functional protein as reported [31]. Subsequently, ELISA analysis of H3K27me3 was used to evaluate the activity of PRC2 in the cells; the results obtained showed that BR0063 was able to effectively reduce the cellular H3K27me3 level in all tested cell lines in a dose-dependent manner, with EC_50_ values of ~1–2 nM (Fig. 1C).

The anti-proliferative effect of BR0063 was also validated for the selected SWI/SNF mutation tumor cell lines through an examination of cell viability, with the cells being incubated with BR0063 serial diluted from 1 μM, as well as DMSO control. Among the nine cell lines tested, two of them (DMS 114 and MFE-296) responded to BR0063 in a dose-dependent manner, with EC50 values of ~10 nM, and a maximum inhibition rate of 80–90% on day 14 of incubation with the compound. Another two cell lines (TOV-112D and HuTu-80) also responded to BR0063, with EC50 values of ~100 nM, although these exhibited a maximum inhibition rate of nearly 100%. The remainder of the SWI/SNF mutation cell lines did not respond to BR0063 up to a concentration of 1 μM (Fig. 1D). It is noteworthy that the inhibitory effect on cell proliferation did not become obvious until after 7 days of BR0063 treatment; such a slow response, however, has also been reported for other PRC2 inhibitors [16].

The results of the proliferation assay suggested that inhibition of PRC2/EED affected the growth of some, but not all, SWI/SNF LOF solid tumor cell lines. Indeed, when EED dependency was investigated from the DepMap database (https://depmap.org/) for the SWI/SNF LOF solid tumor (lung, endometrium and ovary) cell lines, only 17% of the SWI/SNF LOF tumors were found to be sensitive to EED knockdown (RNAi score <-0.2) compared with ~6% for SWI/SNF wild-type tumors (Supplementary Fig. S1), suggesting that SWI/SNF LOF tumors are generally more responsive to PRC2 perturbation; however, the current lack of more precise biomarkers remains an obstacle.

### PRC2 inhibition regulates a common group of genes in the treatment-sensitive cells

**Fig. 2:**
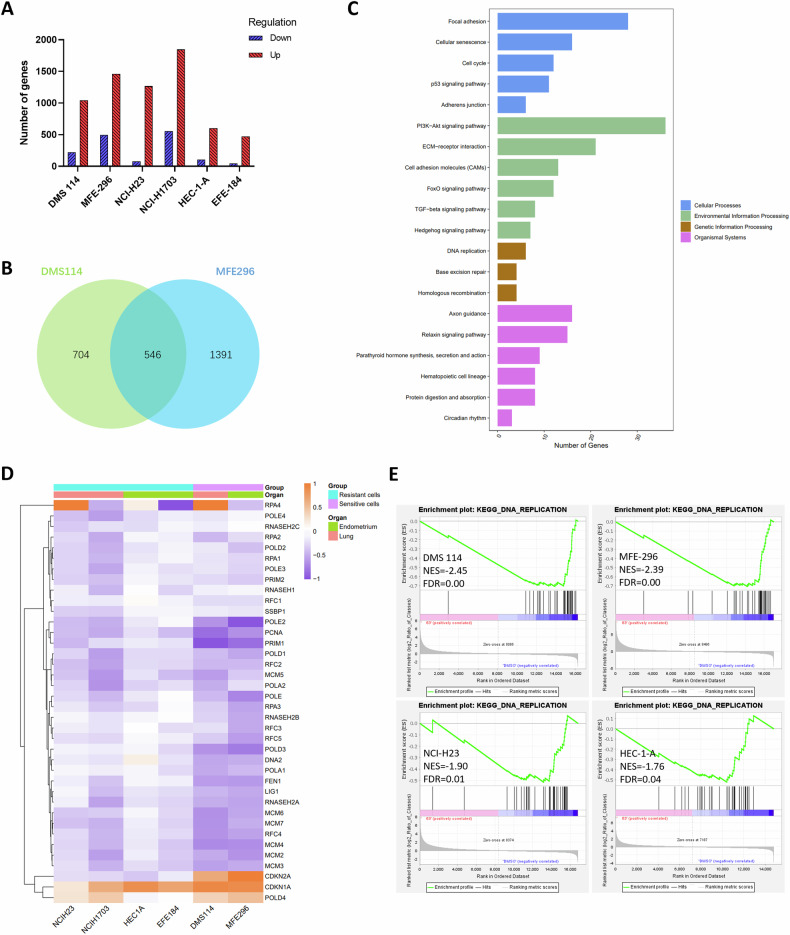
Summary of RNA-Seq data for DMSO and BR0063-treated SWI/SNF LOF tumor cells. **A** The number of genes significantly down- or up-regulated (fold change >1.5 and FDR < 0.05) in BR0063-treated SWI/SNF LOF cell lines compared with the DMSO group for 6 days of treatment are shown. **B** A Venn diagram showing the 564 commonly up- or down-regulated genes identified upon incubation of the sensitive cell lines DMS 114 and MFE-296 with BR0063. **C** KEGG analysis of the 564 commonly up-and down-regulated genes in the DMS 114 and MFE-296 cells demonstrates that cellular senescence, cell cycle, and DNA replication pathways are commonly regulated via PRC2/EED inhibition. **D** Heatmap analysis for Log2 fold changes (BR0063-treated vs. DMSO-treated) of differentially expressed genes in the KEGG DNA replication pathway and of selected genes in the KEGG cell cycle pathway, with purple implying decreased expression while orange implies increased expression. **E** GSEA analysis of Log2 fold changes (BR0063-treated vs. DMSO-treated) of differentially expressed genes in the KEGG DNA replication pathway in DMS 114, MFE-296, HuTu-80, and TOV-112D cells. NES means normalized enrichment score.

To identify factors underlying the PRC2/EED inhibitor responsiveness, RNA-Seq analysis was performed for the control and BR0063-treated SWI/SNF LOF tumor cell lines (i.e., the sensitive cell lines DMS 114 and MFE-296, and the resistant cell lines NCI-H23, NCI-H1703, HEC-1-A, and EFE-184). Most significantly changed genes (fold change >1.5 and FDR < 0.05) were found to be up-regulated in the BR0063-treated samples, which was in accordance with the role of PRC2 in the epigenetic suppression of gene expression (Fig. 2A). A total of 546 commonly regulated genes (471 commonly up- and 75 commonly down-regulated genes) were identified in the two sensitive cell lines (Fig. 2B), and Kyoto Encyclopedia of Genes and Genomes (KEGG) analysis revealed that the top 30 enriched pathways included cell senescence, cell cycle and DNA replication (Fig. 2C). By contrast, the resistant cell lines were also found to share 200–400 commonly up-regulated genes with the sensitive cell lines, but only few of the commonly down-regulated genes (at most, <10) were shared. Further inspection of commonly regulated genes revealed that CDKN2A appeared to be the key factor that is up-regulated in the sensitive cell lines (Fig. 2D), and this has also been reported in other tumor types [16, 32]. Reverse-transcription quantitative PCR (RT-qPCR) (Supplementary Fig. S2) analysis revealed that the transcripts encoding p16, but not p14ARF (denoted as ‘p14’ for short), were upregulated in the tested BR0063-sensitive cells. p16 is known to inhibit cyclin-dependent kinases-4/-6 (CDK4/6)-cyclin D1 (CCND), leading to the accumulation of unphosphorylated retinoblastoma (Rb) protein that further inhibits E2F transcription activity [33]. Indeed, some of the commonly down-regulated genes in sensitive cells were shown to be genes downstream of E2F, including *PCNA*, *PRIM1*, *MCM2, MCM4,* and *ORC1*, which are associated with the cell cycle and DNA replication [34] (Fig. 2D). GSEA was also performed for each cell line, and the results obtained also suggested that “DNA replication”, as a process, was significantly enriched in BR0063-treated sensitive cells (Fig. 2E). Interestingly, the same pathway was also enriched in the resistant cells, albeit with non-significant fold changes. This may be due to the fact that the upregulation of *CDKN1A*/p21 was held in common among the tested cells (Fig. 2D), which also causes inhibition of the downstream gene expression of E2F to a certain extent, but not sufficiently so to affect the overall cell proliferation.

Pathways for commonly regulated genes in both sensitive and resistance cell lines were also inspected, and the results obtained suggested that genes associated with the extracellular matrix (ECM) were mostly upregulated in the BR0063-treated cells (Supplementary Fig. S3A). Certain of these ECM genes are also included among the senescence-associated secretory phenotype (SASP) genes. Indeed, numerous SASP genes were found to be up-regulated upon incubation with BR0063, despite there being no p16 up-regulation or inhibition of cell proliferation (Supplementary Fig. S3B), findings that were in accordance with a recent study [32].

### Inhibition of PRC2 induces cell senescence or apoptosis in the sensitive cells

**Fig. 3:**
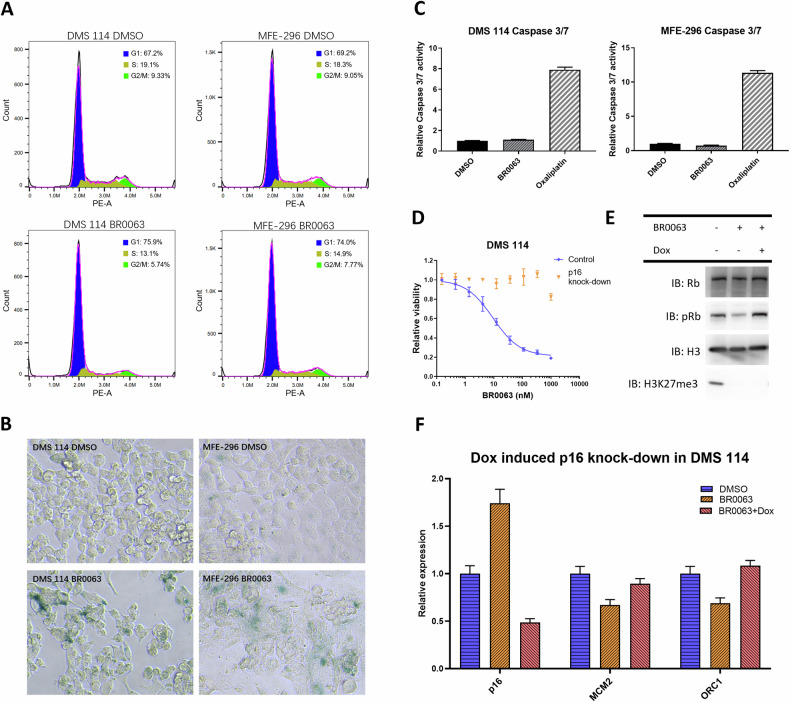
BR0063 inhibits proliferation in DMS 114 and MFE-296 cells by p16-induced cell senescence. **A** Flow cytometry analysis for cell cycle distribution of control and BR0063-treated DMS 114 and MFE-296 cells by PI staining. The result of one representative assay from two similar independent experiments is shown, with *x*- and *y* axes denote PI signal and cell number count, respectively. Only intact and single cells are included in the analysis. Cell population fitted in G1 phase are marked as blue, S phase as yellow, G2/M phase as green. The distribution of the sum of the fitted cell population shown as magenta overlaps with the original distribution shown as black. **B** β-galactosidase staining of DMSO- or BR0063-treated DMS 114 and MFE-296 cells (at ×200 magnification). **C** Caspase-3/7 assay detection of apoptosis in control and BR0063-treated DMS 114 and MFE-296 cells, with the addition of positive control, oxaliplatin, which is known to induce cellular apoptosis. Each experiment was performed in triplicate, and error bars are shown as the mean ± SD. **D** CellTiter-Glo^®^ detection of cell viability of DMS 114 (with or without Dox-induced p16 knockdown) incubated with serial dilution of BR0063 for 14 days, normalized with DMSO control. Each experiment was performed in triplicate, and error bars are shown as the mean ± SD. **E**, **F** WB and qPCR analyses of DMS 114 cells treated with control or BR0063, with or without Dox-induced p16 knockdown. For the qPCR analyses, each sample was run in triplicate, and error bars represent the mean ± SD.

To validate the mechanism of action for BR0063-induced growth inhibition in the PRC2/EED inhibitor-sensitive DMS 114 and MFE-296 cells, cell cycle analysis was performed using PI staining and flow cytometry. These experiments revealed that the cell population percentages were reduced in S and G2/M phases in BR0063-treated cells (Fig. 3A and Supplementary Fig. S4), in agreement with the compound-induced downregulation of genes associated with DNA replication. Since treatment of the DMS 114 and MFE-296 cells with BR0063 also leads to an up-regulation of the expression of p16, which is known to induce cell senescence, the control and BR0063-treated cells were subjected to β-galactosidase staining assay. BR0063-treated cells exhibited a senescent and aged phenotype with a flat and enlarged shape, and the presence of strong blue staining indicated high senescence-associated β-galactosidase activity, which is the marker of senescent cells (Fig. 3B). Since no obvious cell death was observed, the cell apoptotic status was assessed using caspase-3/-7 assay. Compared with the positive control compound oxaliplatin, which is known to induce cell apoptosis, incubation with BR0063 led to no significant change in the caspase signal, suggesting that BR0063 did not induce apoptosis in the DMS 114 and MFE-296 cells (Fig. 3C). This finding also accounted for why the cell viability remained at the ~10–20% level for cells treated with 1 μM BR0063 for 14 days compared with the DMSO control group (Fig. 1D, cell viability assay for DMS 114 and MFE-296).

To further confirm the key role of p16 up-regulation on BR0063-induced senescence and growth inhibition, an shRNA was constructed for the Tet-inducible knockdown of p16 by the stable transfection in the DMS 114 and MFE-296 cell lines. The addition of doxycycline (abbreviated as Dox, cat. no. #A426815, Sangon Biotech) induced knockdown of p16, and completely or partially countered the anti-proliferative effect induced by BR0063 in the DMS 114 and MFE-296 cells, respectively (Fig. 3D and Supplementary Fig. S5A). WB and qPCR analysis showed that p16 knockdown efficiently reversed the up-regulation of p16 expression mediated by incubation with BR0063 and restored Rb phosphorylation, as well as the expression of genes downstream of E2F, including *MCM2* and *ORC1* (Fig. 3E, F, and Supplementary Fig. S5B, C), in both of the tested cell lines.

In contrast with the results with the DMS 114 and MFE-296 cell lines, the results from the β-galactosidase staining and caspase-3/-7 assays demonstrated that BR0063 inhibited cell proliferation by inducing apoptosis, rather than senescence, in the TOV-112D and HuTu-80 cells (Supplementary Fig. S6A, B), a finding that also accounted for the near-100% inhibition rate of cell viability at the higher compound dose (Fig. 1D, cell viability assay for TOV-112D and HuTu-80). In accordance with the phenotype, p16 up-regulation was not observed in these two cell lines (Supplementary Fig. S2A). Although p14 is up-regulated in HuTu-80 cells (Supplementary Fig. S2B), in the present study, we demonstrated that its knockdown did not affect the sensitivity of HuTu-80 cells to treatment with BR0063 (Supplementary Fig. S7). PRC2 inhibition-induced apoptosis in these two cell lines may be mediated by factors other than CDKN2A.

### Hypermethylation of the CGI promoter leads to an inhibition of p16 expression and resistance to BR0063

Since the up-regulation of p16 is a critical factor for BR0063-induced senescence and anti-proliferation, we next considered the underlying cause of resistance to PRC2/EED inhibition in the tested cell lines. Through checking the expression profiles of the resistant NCI-H23 and HEC-1-A cell lines (Supplementary Fig. S2A), it was observed that p16 was hardly expressed in the absence or presence of BR0063. Since neither of the cell lines harbored deletion mutations of *CDKN2A* gene, this indicated that factors other than PRC2 must be responsible for the repression of p16 expression.

**Fig. 4:**
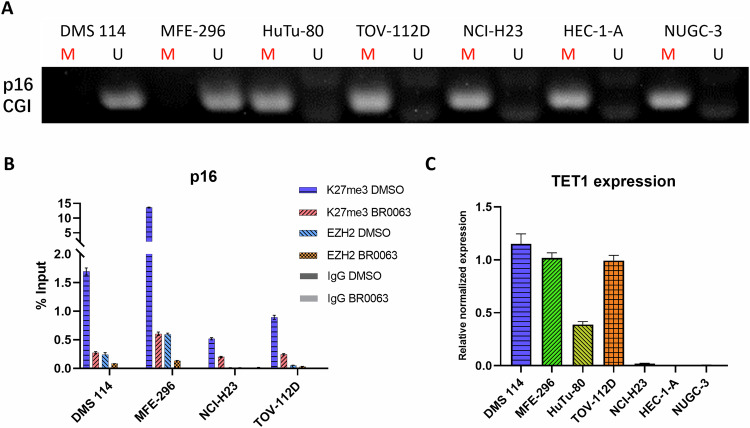
DNA methylation on the p16 CGI promoter is reversely correlated with PRC2 occupancy. **A** Methylation-specific PCR analysis on the p16 CGI promoter region. M means PCR product for methylated CGI region, U means PCR product for unmethylated CGI region. **B** ChIP-qPCR analysis on the occupancy of H3K27me3 and EZH2 at the p16 CGI region of DMS 114, MFE-296, NCI-H23, and TOV-112D cells with or without BR0063 incubation. **C** The expression level of *TET1* in the tested SWI/SNF LOF tumor cells analyzed by qPCR, each sample was run in triplicate, and the error bars represent the mean ± SD.

DNA hypermethylation on CGI is a well-established mechanism for gene silencing [35]. To investigate any correlation between the DNA methylation status of the p16 CGI promoter and the gene expression level, methylation-specific PCR was performed using genomic DNA extracted from selected SWI/SNF LOF tumor cell lines. The results obtained showed that the BR0063-sensitive DMS 114 and MFE-296 cells had a low level of DNA methylation at the p16 CGI promoter, their basal p16 expression is higher, and respond to BR0063 treatment with obvious up-regulation; whereas BR0063-resistant cells with high methylation in this region had a very low expression level of p16, and mostly did not respond to BR0063 treatment (Fig. 4A and Supplementary Fig. S2A). Taken together, these results suggested that DNA hypomethylation on the p16 CGI promoter is the key factor for BR0063-induced p16 up-regulation and cell senescence. The occupancy of the repressive histone marker H3K27me3, as well as of the PRC2 subunit EZH2 at the p16 promoter region, was also measured by chromatin immunoprecipitation (ChIP)-qPCR analysis (Fig. 4B). Strong occupancy of H3K27me3 and EZH2 was identified in the DMS 114 and MFE-296 cells, and was clearly decreased following treatment with BR0063, suggesting that it is the removal of the PRC2 complex, in addition to repression marks in this genomic region upon EED perturbation, that leads to the activation of p16 expression. By contrast, NCI-H23 and TOV-112D cells, wherein p16 protein is absent, had a low level of H3K27me3, and an absence of EZH2 occupancy on the p16 promoter region, suggesting that p16 suppression is mainly not mediated by H3K27me3 and PRC2 activity. Taken together, these findings suggest that DNA methylation and occupancy of the H3K27me3 marker are mutually exclusive at the p16 CGI region, and cells with p16 CGI hypermethylation may not depend on PCR2 activity for the suppression of this gene. Interestingly, previous studies have also reported a strong antagonism between DNA hypermethylation and the H3K27me3 histone markers on CGIs throughout the bulk of the genome in both mouse embryonic stem [36] and zebrafish embryos [37], suggesting that an evolutionarily conserved mechanism of gene regulation is in operation here.

To further identify the factors associated with the DNA methylation status of the p16 promoter, the expression of genes associated with DNA methylation across different cells was assessed based on the RNA-Seq data (Supplementary Fig. S8A). Only *TET1* appeared to be differentially expressed in the BR0063-sensitive and -resistant cells, and this was validated by qPCR analysis (Fig. 4C). TET1 is a tumor suppressor that is required for DNA demethylation, which counters the effects of DNA methyltransferase-induced CGI hypermethylation and gene expression suppression [38]. Interestingly, although the TOV-112D/HuTu-80 cells responded to BR0063 inhibition via a completely different mechanism compared with the DMS 114/MFE-296 cells, both groups of cells exhibited a higher expression level of *TET1* compared with the resistant cells, indicating that genes associated with apoptosis in the TOV-112D and HuTu-80 cells may also be silenced through CGI hypermethylation in the resistant cells, similarly to the case of p16, and that *TET1* expression might therefore serve as a common biomarker for the prediction of cellular response to treatment with BR0063.

### BR0063 inhibits tumor growth in DMS 114 and MFE-296 xenograft models

**Fig. 5:**
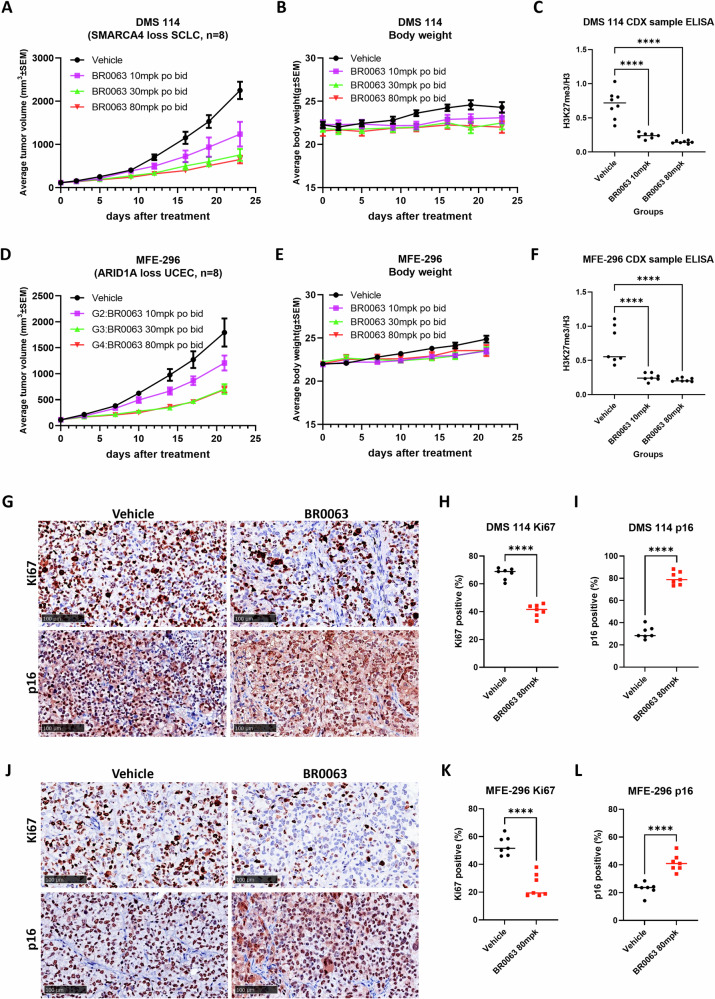
BR0063 demonstrated good in vivo efficacy against cell-derived xenograft model of SWI/SNF LOF mutation solid tumors. **A**, **D** Tumor growth curves of the vehicle and BR0063-treated DMS 114 (**A**) and MFE-296 (**D**) xenograft models. The tumor volume was measured twice a week, calculated by the formula: 0.5×(length × width^2^). BR0063 reduces tumor growth in both models in a dose-dependent manner. **B**, **E** Recorded animal body weights in DMS 114 (**B**) and MFE-296 (**E**) xenograft models. No significant body weight loss was observed at the highest administered dose (80 mpk bid). **C**, **F** ELISA detection on the H3K27me3 marker in the tumor samples from vehicle (*n* = 8) and BR0063 10/80 mpk po bid (*n* = 7, one sample excluded due to limited space of ELISA plate) group of DMS 114 (**C**) and MFE-296 (**F**) xenograft models. The tumor inhibition efficacy was found to correlate with the BR0063-induced depletion of the H3K27me3 marker in tumor samples. **G**–**L** Representative image of IHC staining for the proliferation marker Ki67 (shown as brown chromogen staining of nuclear), and the senescence marker p16 (shown as brown chromogen staining of both nuclear and cytoplasm), in tumor samples of the DMS 114 (**G**) and MFE-296 (**J**) xenograft models. The scale bar represents 100 μm. The relative positive area was calculated, and subsequent statistical analysis was shown in (**H**–**I**) for DMS 114, and (**K**, **L**) for MFE-296. Statistical significance is shown in the figure as follows: *p < 0.05; **p < 0.01; ***p < 0.001; or ****p < 0.0001. Data are presented as scatterplots, with the bars indicating the median value.

Subsequently, in vivo efficacy studies of BR0063 in DMS 114 and MFE-296 xenograft models were performed in female BALB/c nude mice (method schema shown in Supplementary Fig. S9). BR0063 was well tolerated at the highest dose (80 mpk po bid) in both models demonstrated by no loss of body weight (Fig. 5B, E), and clear tumor growth inhibition rates of 75% and 65%, respectively (Fig. 5A, 5D). ELISA detection of the tumor lysate indicated that BR0063 dose-dependently inhibited H3K27me3 in the tumor samples (Fig. 5C, F), suggesting robust on-target activity. The levels of a proliferation marker (Ki67) and the senescence marker, p16, were also investigated in tumor samples by immunohistochemical (IHC) staining (Fig. 5G, I), and these experiments demonstrated that BR0063 significantly (P < 0.0001, Fig. 5H, I, K, L) reduced cell proliferation and induced senescence in both xenograft models that were treated with 80 mpk BR0063, findings that were in agreement with the results obtained in vitro.

## Discussion

PRC2-mediated tri-methylation on histone H3K27 is one of the histone modifications responsible for gene silencing, and such a repressive state can be switched to active transcription by the ATP-dependent chromatin-remodeling complex, SWI/SNF. Loss of homeostasis between these two complexes, either by overactivation of PRC2 or by LOF mutations of SWI/SNF, drives the progression of cancer [11, 12]. LOF of the components of the SWI/SNF complex, mainly ARID1A, SMARCA4, or SMARCB1, has been demonstrated to sensitize cancer cells to PRC2 inhibitors [13, 14, 31]. Considering the high mutation rate of the SWI/SNF complex among different types of solid tumors, PRC2 inhibitors are potentially able to benefit a large patient population. However, in terms of a potential strategy for therapeutic intervention, the clinical efficacy of PRC2 inhibitors in solid tumors remains unsatisfactory [18]. Cancer cells bearing SWI/SNF mutations have often been found to be resistant to PRC2 inhibition, and multiple underlying mechanisms to account for this resistance have been proposed [15, 39]. However, at present, no precise biomarkers have been shown to be able to predict the response of SWI/SNF LOF tumors to PRC2 inhibitors.

In the present study, we have demonstrated that PRC2 inhibition induces cell senescence or apoptosis in a subset of SWI/SNF LOF tumor cells, as well as clear tumor growth inhibition in the corresponding xenograft mouse models. Gene expression and pathway analyses indicated that BR0063-induced senescence was primarily due to the up-regulation of *CDKN2A*/p16, followed by a decrease in the level of Rb phosphorylation and expression of genes downstream of E2F, which caused deficiencies in DNA replication and cell-cycle arrest. Interestingly, data from the DepMap database for *CDKN2A* copy numbers and EED dependency in the lung, endometrial, and ovarian tumor cells indicated that the *CDKN2A* deletion cells (copy number <1) are generally not responsive to EED perturbations (Supplementary Fig. S10A), suggesting that *CDKN2A* copy number loss may serve as a reference biomarker for the resistant cells. Apart from copy number loss, the suppression of p16 expression may also result from CGI hypermethylation; such suppression cannot be reversed by PRC2 inhibition, and this alternative process may cause cell resistance to BR0063-induced senescence. This hypothesis could also be supported by the DepMap data, which demonstrated that solid tumor cells with a low fraction of p16 CGI methylation are more sensitive to EED perturbation, compared with the cells of the types that did have p16 CGI hypermethylation (Supplementary Fig. S10B).

Although we have not identified the exact genes responsible for BR0063-induced apoptosis in the TOV-112D and HuTu-80 cells, the RNA-Seq data for TOV-112D revealed the up-regulation of multiple tumor suppressor genes that could promote apoptosis, including *CDKN1A* and *GADD45A* (Supplementary Fig. S8B). It is worth noting that, even for the cells with BR0063-induced senescence, p16 is not the only factor that caused growth inhibition, as p16 knockdown in MFE-296 cells could not completely reverse the anti-proliferative effect of BR0063 (Supplementary Fig. S5A). The apoptotic and senescent phenotypes induced by BR0063 may result from multiple factors and processes regulated by PRC2.

**Fig. 6:**
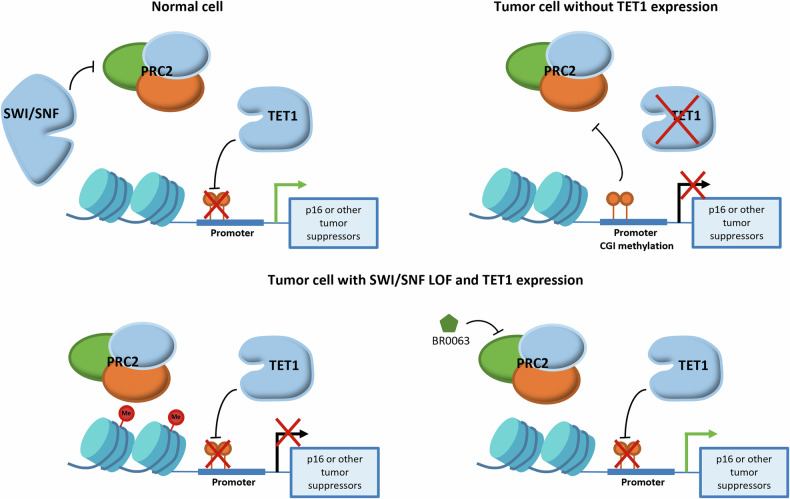
Proposed mechanism of correlation between *TET1* expression, CGI methylation, and PRC2 regulation on tumor suppressors, including p16. In normal cells, the function of PRC2 is regulated by the SWI/SNF complex, whereas in tumor cells featuring SWI/SNF LOF mutations in addition to *TET1* expression, the recruitment of PRC2 on the p16 promoter is allowed to occur, and suppression of its expression by the H3K27me3 marker is mediated.

Epigenetic regulation, including DNA methylation and histone modification, influences gene expression at the transcriptional level. The present study has demonstrated the antagonism that exists between PRC2 regulation and the CGI methylation-mediated suppression of p16, which led us to consider that one or the other suppression mechanism may also be operative for genes, including other tumor suppressors. Indeed, the present study has suggested that the absence of TET1, an active 5mC hydroxylase that hydroxylates 5mC to 5hmC and initiates the DNA demethylation process, appears to correlate with both low PRC2 occupancy on the p16 promoter and resistance to BR0063 in our tested SWI/SNF LOF cell lines. The DepMap analysis also revealed that a high *TET1* expression correlated with low CGI methylation on the p16 promoter (Supplementary Fig. S10C), which resulted in a higher level of EED dependency (Supplementary Fig. S10D). Interestingly, TET1 has been reported to co-localize with PRC2 on the gene promoter region, and this was required for the recruitment of PRC2 to CpG-rich gene promoters [40]. Based on the above evidence, we hypothesized that TET1 is able to reduce the level of DNA methylation on the CGI promoter of genes, including tumor suppressors, thereby allowing the recruitment of PRC2, which effectively switches the mechanism of regulation of gene expression from DNA methylation to an activated H3K27me3 histone marker (Fig. 6).

Based on the findings of the present study, we propose the following additional criteria for the prediction of the PRC2 inhibition response in SWI/SNF LOF tumors: No *CDKN2A* gene deletion or copy number loss (*CDKN2A* average copy number > 1), and no silencing of *TET1* expression (positive *TET1* expression, as indicated by TPM > 1). We applied these new selection criteria in the analysis of the DepMap EED dependency score, and found that 20 of 41 SWI/SNF LOF lung/endometrium and ovary tumor cell lines met with these conditions, and seven of them were EED-dependent, yielding response rates of ~35%, greatly improved on that of the unselected cell lines (~17%; 7 in 41 cells; see Supplementary Fig. S2B). Moreover, these selection criteria had the further advantage of retaining the entire population of sensitive cells, only excluding the resistant cells. The selected sensitive cells included the cells validated by BR0063 treatment in the present study (DMS 114 and MFE-296 in Fig. 1D, and NCI-H841 in Supplementary Fig. S11), as well as cell lines validated by treatment with other PRC2 inhibitors in previous studies (e.g., NCI-H522 [15, 41]). Even for the ‘resistant’ cells (RNAi score >-0.2) that fit with the selection criteria, several of these are actually sensitive cells, such as the TOV-112D (Fig. 1D), TOV-21G and COV434 cell lines [15, 41], suggesting that the real response rate would be even higher. The analysis using the EZH2 dependency scores gave rise to similar results (data not shown). Interestingly, for cells with no SWI/SNF mutation, applying these selection criteria also led to an increase in the EED/EZH2 dependency rate (Supplementary Fig. S2), suggesting that this selection method could apply to tumors with high PRC2 activity induced by factors other than SWI/SNF LOF mutations, such as the overexpression of EHZ2.

Although the selection criteria are appropriate for the prediction of PRC2 inhibition-sensitive cell lines from endometrium, lung, and ovary tumors so far, we understand such a hypothesis needs to be validated using more cell lines and xenograft models. In addition, there are limitations in the prediction of PRC2 inhibition-sensitive cancer cells of gastrointestinal and breast origin using the current strategy. Indeed, although PRC2 regulates 500-1000 genes in the tested cells (Fig. 2A), PRC2-regulated genes mostly do not overlap in different cancer cell lines, which means PRC2 may regulate completely different sets of genes/pathways in cancer cells of diverse lineage. Thereby, a more precise set of biomarkers is required for further investigations of the role of PRC2 in these cell lines.

In conclusion, the present study has demonstrated that *CDKN2A*/p16 up-regulation is the key factor for BR0063-induced senescence, and such regulation is abolished by DNA hypermethylation on the CGI region of the gene, which excludes the PRC2 complex and H3K27me3 markers from this region. The expression of *TET1* appears to correlate with the DNA methylation status on CGI, and this may, therefore, serve as a biomarker for the prediction of responsiveness of SWI/SNF LOF tumors to PRC2/EED inhibitors. Taken together, these findings may provide more precise biomarkers for the selection of patients with solid tumors bearing SWI/SNF LOF mutations that would be able to benefit from PRC2 inhibitor treatment.

## Supplementary information

Supplementary information

Original WB data

## Data Availability

Plasmids, proteins, and peptides are available upon reasonable request. RNA-seq data have been deposited at CNSA with accession number CNP0005260. This paper does not report the original code. Uncropped western blots are available in the Supplemental Material. Any additional information required to reanalyze the data reported in this paper is available from the corresponding author upon request.
