# Supplementary material for: Hypermethylation of CDKN2A CpG island drives resistance to PRC2 inhibitors in SWI/SNF loss-of-function tumors
Source: Cell Death Dis. 2024 Nov 5;15(11):794. doi: 10.1038/s41419-024-07109-3 (PMC11538500; doi:10.1038/s41419-024-07109-3)
Supplement: Supplementary file 1 — Supplementary information [file 41419_2024_7109_MOESM1_ESM.pdf]

Supplementary information

Supplementary table 1. SWI/SNF mutation status (from COSMIC and CCLE database) in selected solid tumor cell lines.

Cell lines	Cancer type	SWI/SNF alternation
**NCI-H23**	Lung (Adeno)	SMARCA4 p.E1567*
**NCI-H1703**	Lung (Squamous)	SMARCA4 Splice_Site, SNP
**DMS 114**	Lung (SCLC)	SMARCA4 p.E1310*
**MFE-296**	Endometrium	ARID1A p.M274fs
**TOV-112D**	Endometrioid carcinoma of ovary	SMARCA4 p.L639fs*7
**HEC-1-A**	Endometrium	ARID1A p.Q1835* p.Q2115* DEL
**EFE-184**	Endometrium	ARID1A p.S552fs
**HuTu-80**	Small intestine	SMARCA4 p.K635fs*9
**NUGC-3**	Gastric	ARID1A p.D1850Gfs*4

Supplementary table 2. List of primers used in this study.

**Primers**	
ACTB-F	CAAAGTTCACAATGTGGCCGAG
ACTB-R	TTAGAGAGAAGTGGGGTGGCT
p16-F	TCGGGTAGAGGAGGTGCG
p16-R	GGCCTCCGACCGTAACTATT
p14-F	GGGTTTTCGTGGTTCACATCC
p14-R	CTAGACGCTGGCTCCTCAGTA
ORC1-F	ACCGAGATTCACATCCAGATTGG
ORC1-R	CGAGCACGTTTCTTAGGAGGA
MCM2-F	ATGGCGGAATCATCGGAATCC
MCM2-R	GGTGAGGGCATCAGTACGC
TET1-F	CATCAGTCAAGACTTTAAGCCCT
TET1-R	CGGGTGGTTTAGGTTCTGTTT
p16-ChIP-F	GGCCTCCGACCGTAACTATTC
p16-ChIP-R	TCGGGTAGAGGAGGTGCG
p16-MF	TTATTAGAGGGTGGGGCGGATCGC
p16-MR	GACCCCGAACCGCGACCGTAA
p16-UF	TTATTAGAGGGTGGGGTGGATTGT
p16-UR	CAACCCCAAACCACAACCATAA

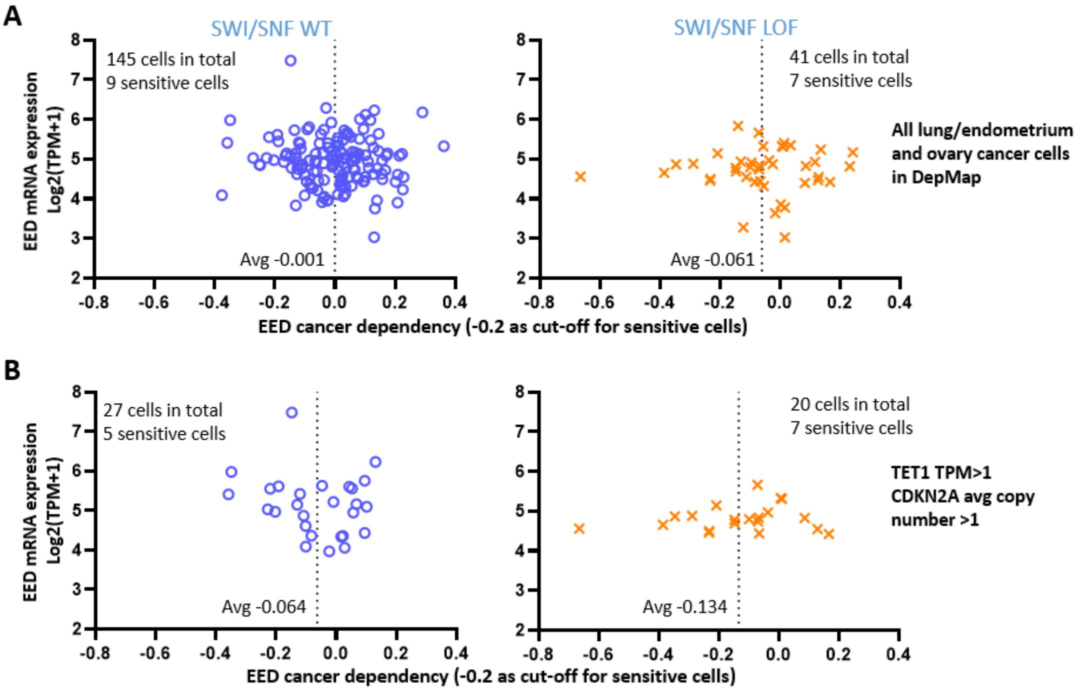

Supplementary Figure 1. DepMap EED dependency analysis for SWI/SNF wild-type and LOF mutation cells. (A) Analysis of EED dependency in SWI/SNF LOF and wild-type (WT) solid tumor cells (lung, endometrium and ovary) showing that EED is dependent (gene-effect RNAi score <−0.2) in less than one-fifth of the SWI/SNF LOF cells; therefore, the SWI/SNF mutation status alone may not be an accurate biomarker for the tumor's response to PRC2 inhibition. (B) Application of additional biomarkers, e.g., no CDKN2A gene deletion or copy number loss (CDKN2A average copy number>1), and no silencing of TET1 expression, greatly improved the dependency rate of EED in the SWI/SNF LOF tumor cells.

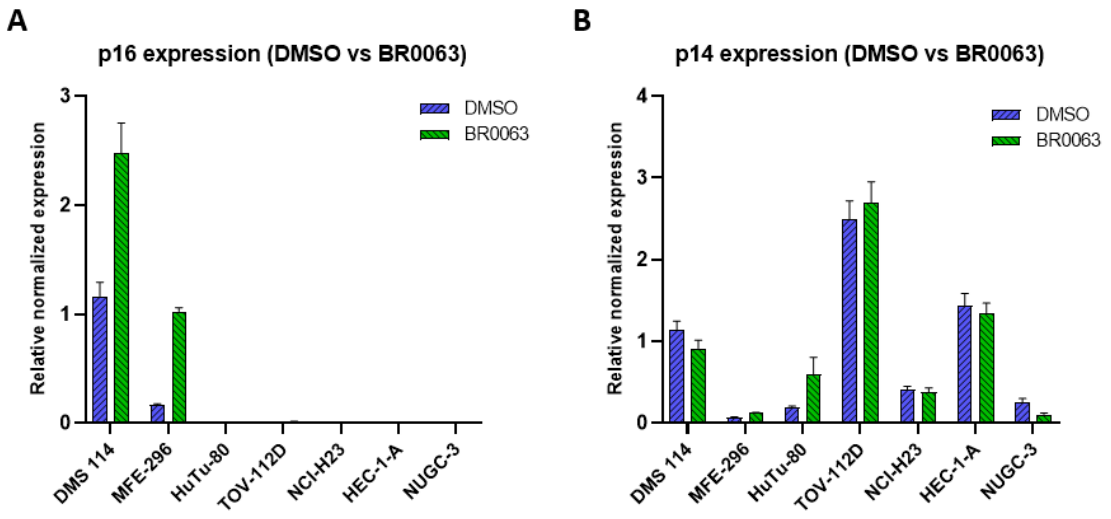

Supplementary Figure 2. Relative expression of p16 or p14 in SWI/SNF LOF tumor cells. Relative expression (BR0063 treated vs. DMSO control) of (A) p16 or (B) p14 in SWI/SNF LOF tumor cells is shown. Assessment of the relative expression of p16 or p14 in SWI/SNF LOF tumor cells determined that EED inhibitor-sensitive DMS 114 and MFE-296 cells have basal p16 expression, as well as up-regulation of p16 via PRC2 inhibition, whereas p14 expression was not generally affected. For qPCR analysis, each sample was performed in triplicate, and error bars are shown as the mean ± SD.

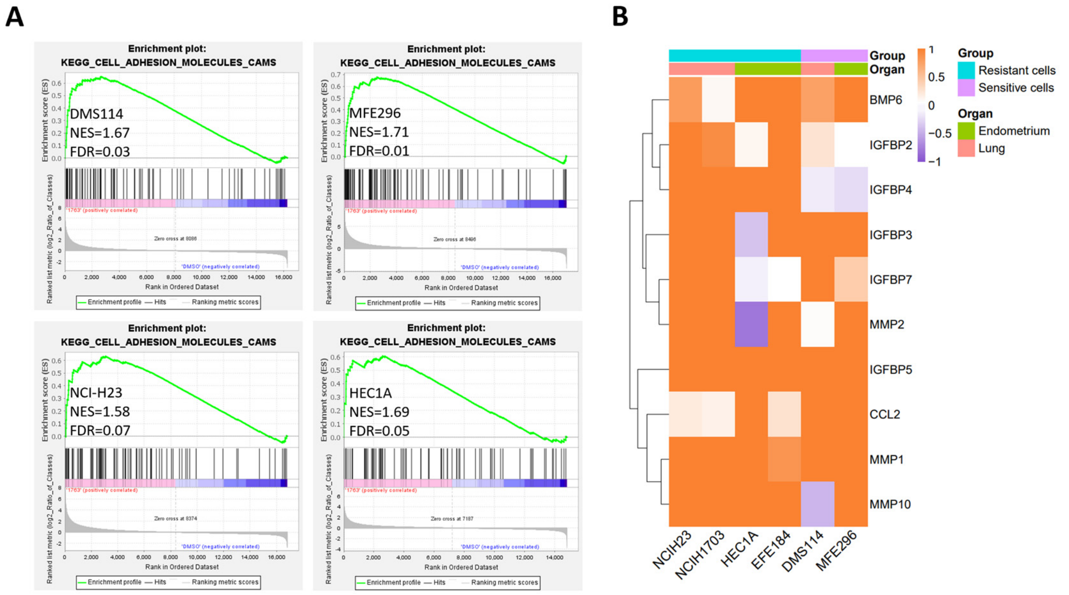

Supplementary Fig. 3. PRC2 inhibition leads to the up-regulation of senescence-associated secretory phenotype (SASP) genes. (A) Gene Set Enrichment Analysis (GSEA) analysis of genes associated with the extracellular matrix (ECM) indicates they are generally up-regulated in the BR0063-treated cells, irrespective of the inhibitory compound's influence on cell proliferation. (B) Heatmap analysis of fold changes of selected SASP genes (BR0063-treated vs. DMSO), showing the up-regulation of SASP genes via PRC2 inhibition. The color scale was used to represent the $\log_2$FC values.

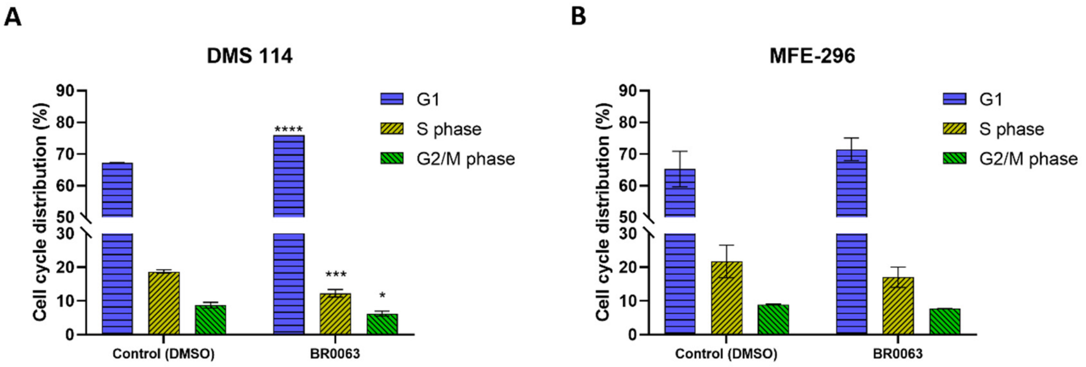

Supplementary Figure 4. Statistical analysis on the percentage of population in each cell cycle phases in control (DMSO) or BR0063-treated DMS 114 (A) and MFE-296 (B) cells. Each experiment was performed in duplicate, and the error bars are shown as the mean ± SD. Statistically significant values are indicated as follows: $^{*}p<0.05$; $^{**}p<0.01$; $^{***}p<0.001$; and $^{****}p<0.0001$.

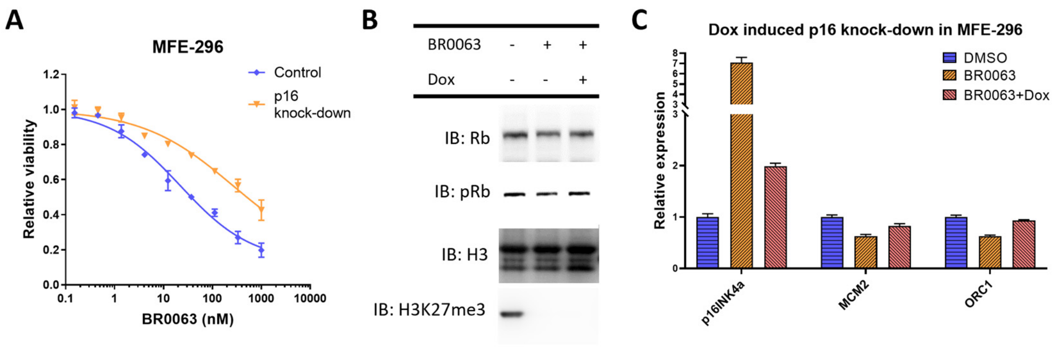

Supplementary Figure 5. BR0063 inhibits proliferation of the MFE-296 cells through the up-regulation of p16. (A) Knockdown of p16 in MFE-296 cells reduced the anti-proliferative effect of BR0063, and the EC_{50} value for control cells (w/o Dox) was 22 nM, compared with the value of 264 nM for cells with p16 knockdown by Dox. Each experiment was performed in triplicate, and the error bars represent the mean ± SD. (B and C) WB and qPCR analysis were performed, indicating that treatment with BR0063 in the MFE-296 cells led to a marked reduction in the cellular H3K27me3 level, together with up-regulation of p16, inhibition of Rb phosphorylation and down-regulation of E2F downstream genes MCM2 and ORC1. Dox-induced knock-down of p16 restored Rb phosphorylation and the expression of MCM2 and ORC1in the BR0063-treated cells, the same as was found in DMS 114 cells. For qPCR analysis, each sample was performed in triplicate, and error bars are shown as the mean ± SD.

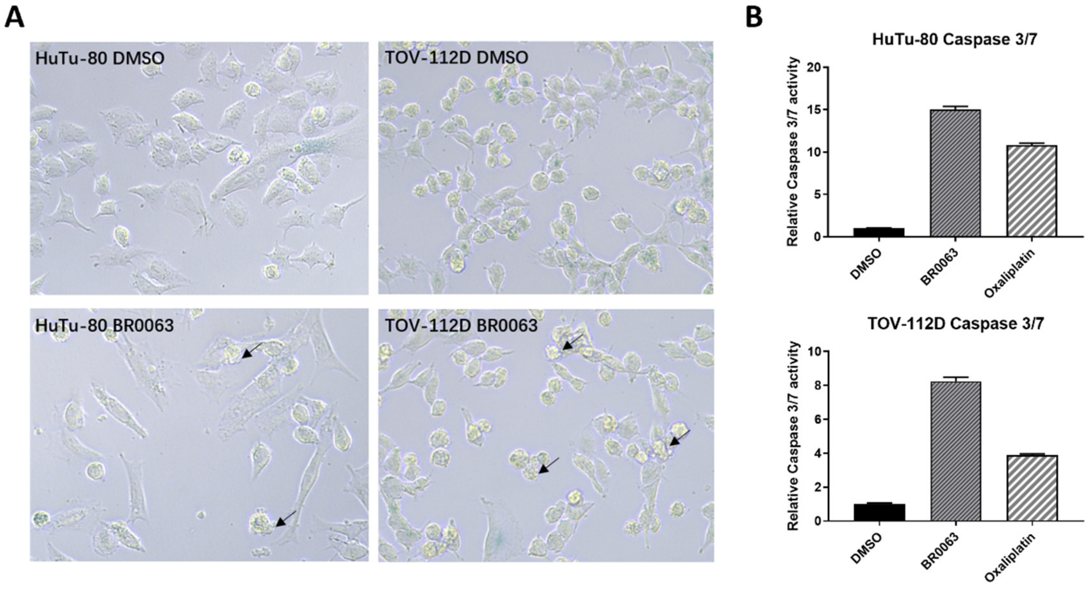

Supplementary Figure 6. Senescence β-galactosidase staining and caspase-3/-7 assay results in BR0063-incubated TOV-112D and HuTu-80 cells. Senescence β-galactosidase staining and caspase-3/-7 assay results for TOV-112D and HuTu-80 cells incubated with BR0063 revealed that PRC2 inhibition induces apoptosis, not senescence, in these two cell lines. (A) β-Galactosidase staining of DMSO- and BR0063-treated HuTu-80 and TOV-112D cells. There was no sign of cellular senescence for BR0063-treated cells, whereas treatment led to the generation of structures similar to apoptotic bodies (indicated in the figure with arrows). (B) BR0063 incubation led to a significant increase in caspase-3/-7 activity in the HuTu-80 and TOV-112D cells. Each experiment was performed in triplicate, and the error bars are shown as the mean ± SD.

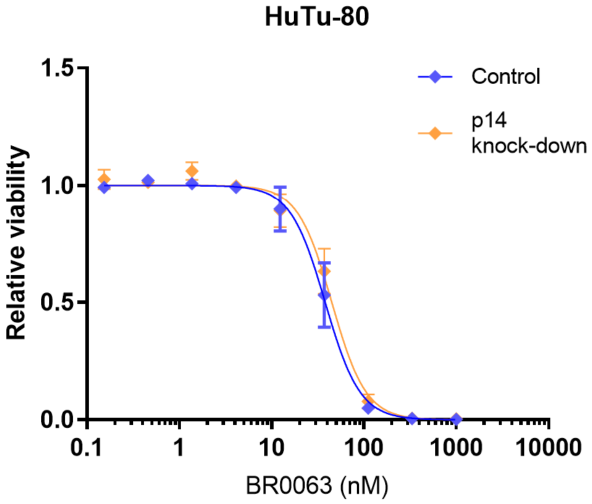

Supplementary Figure 7. Cell viability of HuTu-80 cell incubated with serial dilution of BR0063 in the presence and absence of p14 knock-down. p14 knock-down had no effect on the anti-proliferation of BR0063 in HuTu-80 cell after 14 days of incubation. Each experiment was performed in triplicate, and error bars are shown as the mean ± SD.

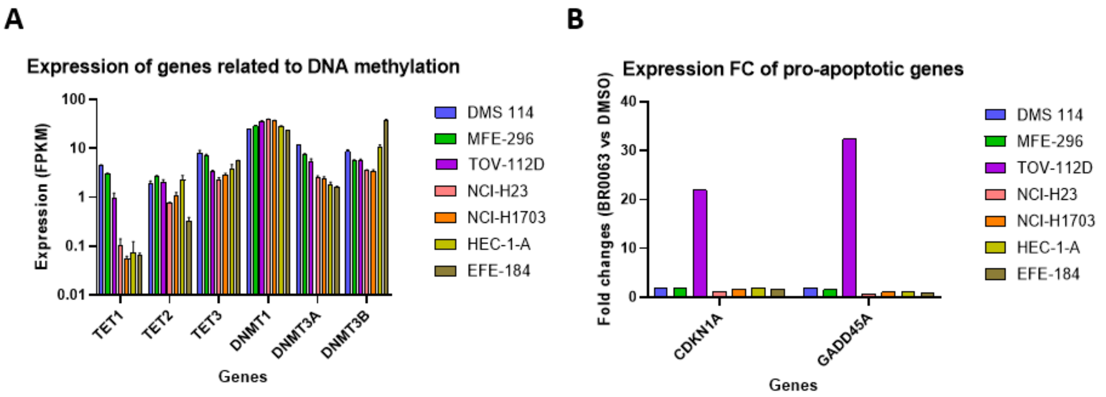

Supplementary Figure 8. RNA-Seq data analysis for genes related to DNA methylation and apoptosis. (A) The expression levels [according to Fragments Per Kilobase Million (FPKM)] of genes associated with DNA methylation across tested SWI/SNF LOF mutation cell lines are shown. The results indicated that only TET1 was differently expressed between the PRC2-inhibition-sensitive (DMS 114, MFE-296 and TOV-112D cells) and PRC2-inhibition-resistant cells. Experiments were performed in duplicate, and the error bars are shown as the mean ± SD. (B) Analysis of fold changes of pro-apoptotic genes (BR0063-treated vs. DMSO-treated) from RNA-Seq data across the tested SWI/SNF LOF mutation cell lines, showing that TOV-112D cells that demonstrated the apoptotic phenotype with PRC2 inhibition exhibited a strong up-regulation of CDKN1A and GADD45A expression.

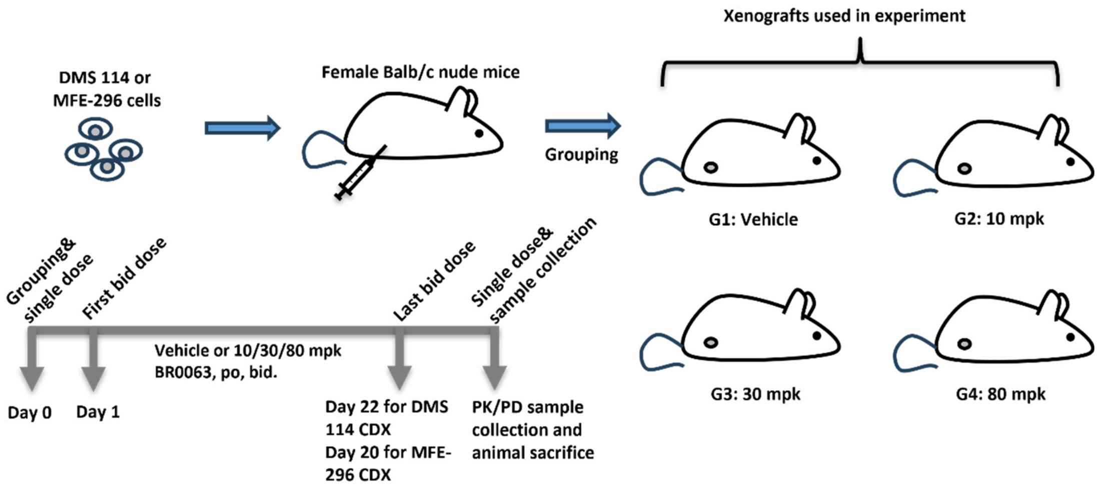

Supplementary Figure 9. Method schema for *in vivo* efficacy studies on DMS 114 and MFE-296 xenograft models.

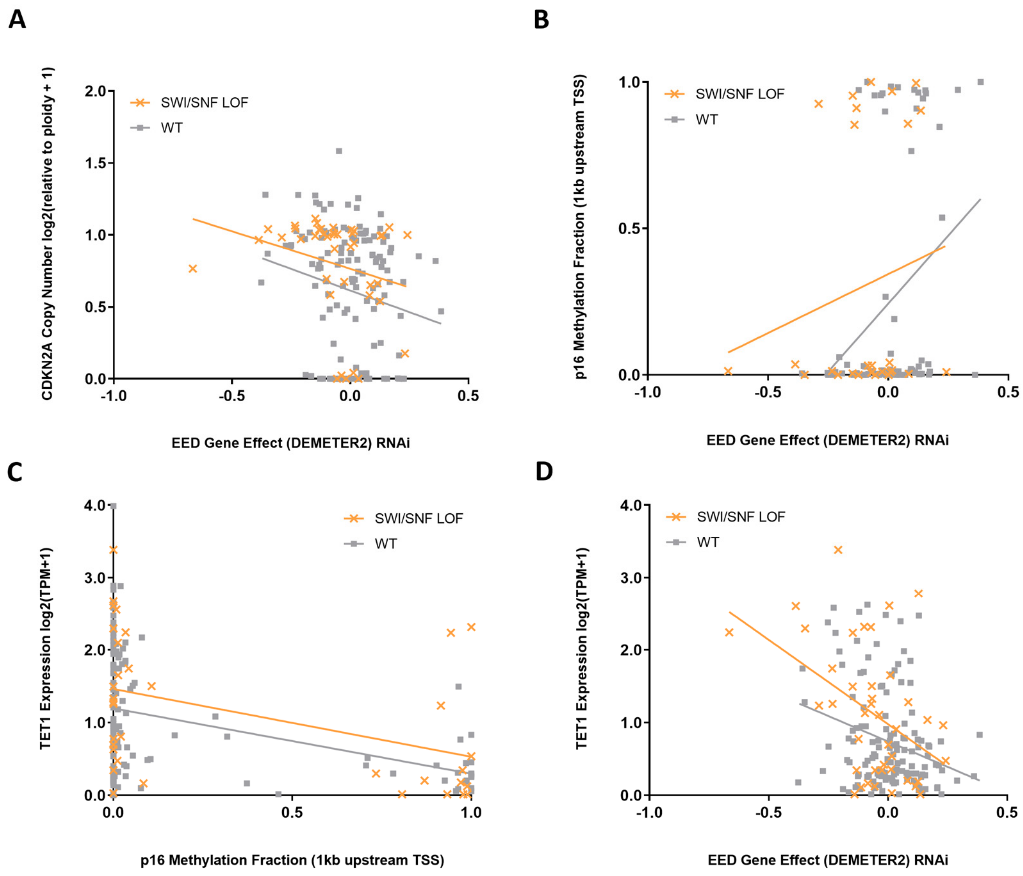

Supplementary Figure 10. DepMap data analysis for correlation between EED dependency, CDKN2A copy number, p16 CGI methylation and TET1 expression in lung, endometrium and ovary cancer cells. DepMap data analysis for correlation between EED dependency, CDKN2A copy number, p16 CGI methylation and TET1 expression in selected types of cancer cells is shown. Cells with SWI/SNF LOF mutations are indicated by asterisks. (A) EED was found not to be dependent (higher Gene Effect score) in selected types of tumors with copy-number loss/deletion of CDKN2A. (B) EED was found to be more dependent in selected types of tumors with low CGI methylation of the p16 promoter. (C) The majority of cells with higher TET1 expression levels (TPM>1) lacked CGI methylation on the p16 promoter. (D) EED was found to be more dependent in selected types of cancer cells with higher expression of TET1.

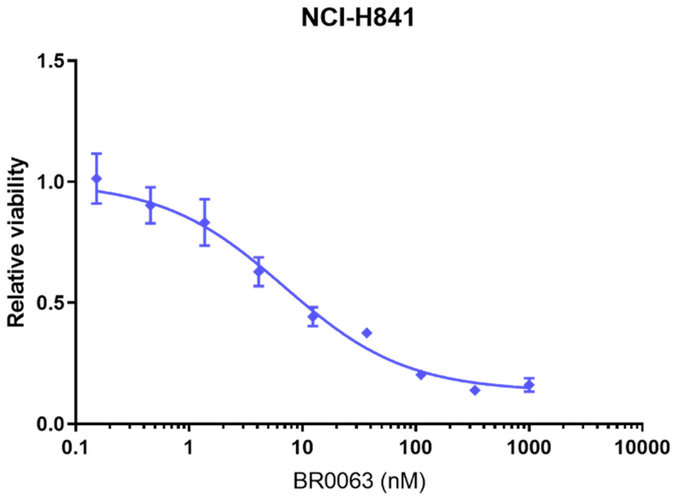

Supplementary Figure 11. Cell viability of NCI-H841 cell incubated with serial dilution of BR0063. BR0063 dose-dependently inhibits the proliferation of NCI-H841 cells with an EC_{50} value of 6.9 nM after 14 days of incubation. Each experiment was performed in triplicate, and error bars are shown as the mean ± SD.
