# Supplementary figures and images for: Hypermethylation of CDKN2A CpG island drives resistance to PRC2 inhibitors in SWI/SNF loss-of-function tumors
Source: Cell Death Dis. 2024 Nov 5;15(11):794. doi: 10.1038/s41419-024-07109-3 (PMC11538500; doi:10.1038/s41419-024-07109-3)

**B**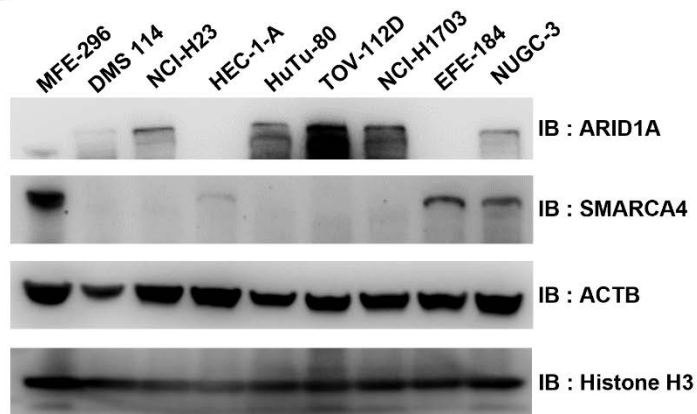**Figure 1****E**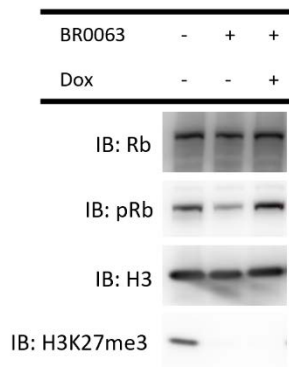**Figure 3**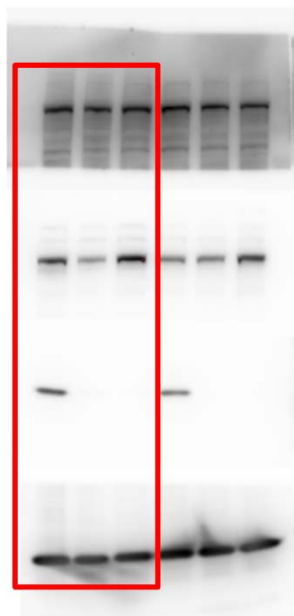

**B**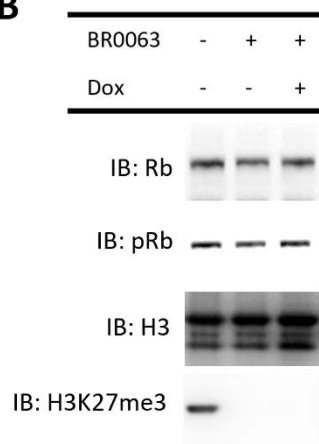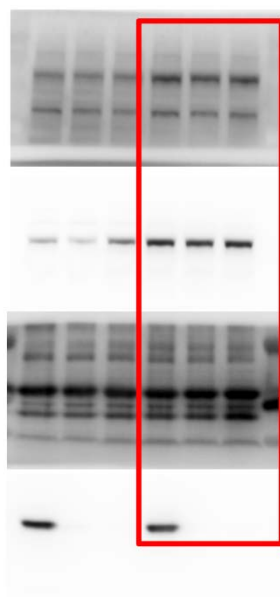

**Supplementary Figure 5**

Supplement: Supplementary file 2 — Original WB data [file 41419_2024_7109_MOESM2_ESM.pdf]
